# The Task Pre-Configuration Is Associated With Cognitive Performance Evidence From the Brain Synchrony

**DOI:** 10.3389/fncom.2022.883660

**Published:** 2022-05-06

**Authors:** Jie Xiang, Chanjuan Fan, Jing Wei, Ying Li, Bin Wang, Yan Niu, Lan Yang, Jiaqi Lv, Xiaohong Cui

**Affiliations:** College of Information and Computer, Taiyuan University of Technology, Taiyuan, China

**Keywords:** synchrony, high-order cognitive networks, the task-general architecture, update efficiency, cognitive performance

## Abstract

Although many resting state and task state characteristics have been studied, it is still unclear how the brain network switches from the resting state during tasks. The current theory shows that the brain is a complex dynamic system and synchrony is defined to measure brain activity. The study compared the changes of synchrony between the resting state and different task states in healthy young participants (*N* = 954). It also examined the ability to switch from the resting state to the task-general architecture of synchrony. We found that the synchrony increased significantly during the tasks. And the results showed that the brain has a task-general architecture of synchrony during different tasks. The main feature of task-based reasoning is that the increase in synchrony of high-order cognitive networks is significant, while the increase in synchrony of sensorimotor networks is relatively low. In addition, the high synchrony of high-order cognitive networks in the resting state can promote task switching effectively and the pre-configured participants have better cognitive performance, which shows that spontaneous brain activity and cognitive ability are closely related. These results revealed changes in the brain network configuration for switching between the resting state and task state, highlighting the consistent changes in the brain network between different tasks. Also, there was an important relationship between the switching ability and the cognitive performance.

## Introduction

The resting state and task state functional magnetic resonance imaging (fMRI) is widely used for the non-invasive assessment of functional brain activity. The recent studies have led to many studies describing the relationship between task state and resting state regions (Friston, 2011; Dajani et al., 2020; Freitas et al., 2020). The recent studies have found that global information transmission and the integration of resting state networks (RSNs) will be more efficient during the mission. The economic theory based on brain network organization indicates that the brain network should be in an energy-saving mode at the resting state, and at the same time showed a dynamic network reorganization under the task requirements to promote the transmission of information between network and network (Bullmore and Sporns, 2012; Avena-Koenigsberger et al., 2017). However, most of them are based on the study of static properties such as the functional connections and the network topology (Markett et al., 2020; Zhang et al., 2020; Zhou et al., 2021), and how the brain network coordinates to express cognitive operations in dynamic properties is still unclear.

The current studies have confirmed that the brain is a complex dynamic system (Vasa et al., 2015; Kringelbach and Deco, 2020), so the brain should be further developed from dynamic perspective research. Synchrony is an important feature of the non-linear dynamics of the brain. In the brain, the communication between different brain regions should take place through coherence, in which two brain regions with synchronous fluctuations of activity can exchange information (Fries, 2005).

Some recent studies advocated a more general architecture between tasks, while other studies advocated differentiation between tasks. We believed that a general architecture between multiple tasks would greatly simplify the research of functional brain organization. This eliminates the need to consider almost infinitely diverse task states and only needs to focus on a single (or a few) network architecture with severely constrained state space. Studies have confirmed that the brain has a general architecture of functional interconnection networks among various tasks, and the functional network architecture during task execution is mainly a RSNs architecture caused by changes in general tasks and task-specific networks (Schultz and Cole, 2016a,b).

We assumed that there was also a task-general architecture of synchrony during the tasks. First, we tried to calculate the synchrony of interaction between different RSNs, exploring the differences between resting state and different tasks. Second, we seek the commonalities of these changes. Finally, we studied the switching ability of synchrony from resting state to task-general architecture and explored the relationship with synchrony and cognitive performance, including the task accuracy, and fluid intelligence, and crystallized intelligence.

## Methods

**Figure 1:**
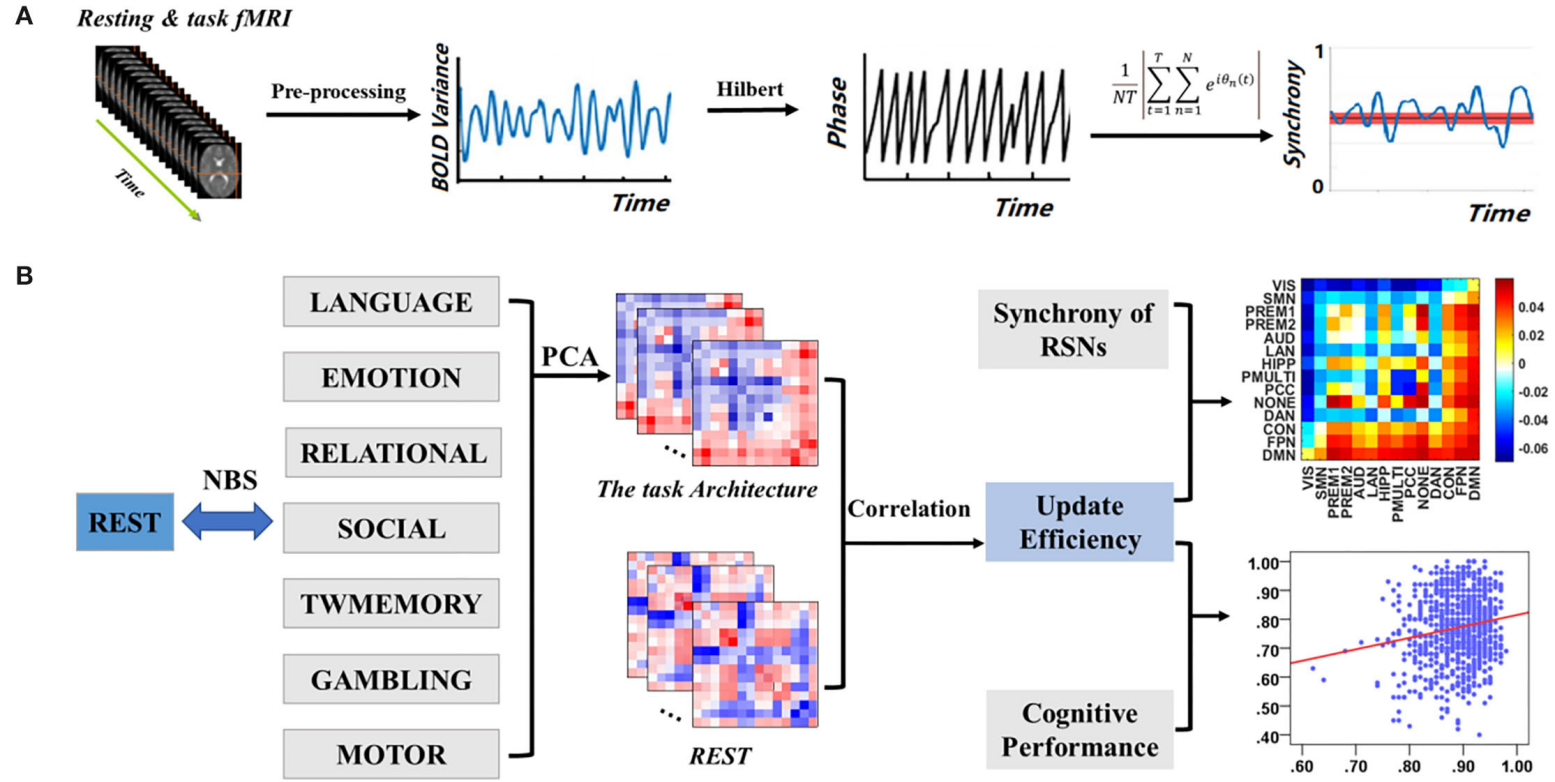
Overview of experimental design. **(A)** Data pre-processing and calculate synchrony. **(B)** Relationship between resting state and task states.

The overall experimental design was shown in Figure 1. The blood oxygenation level-dependent (BOLD) signal was extracted from the pre-processed image and filtered the extracted BOLD signal. Then the BOLD signal was converted into phase through Hilbert transform, and synchrony was calculated (Figure 1A). Subsequently, the synchrony interaction matrix of resting state and task states was verified by network-based statistic (NBS) analysis, and the task-general architecture of synchrony was obtained by principal component analysis (PCA). Finally, a linear regression was performed for update efficiency and synchrony, as well as cognitive performance (Figure 1B).

### Participants

The data were collected by the Washington University–Minnesota Consortium Human Connectome Project (Van Essen et al., 2013). The data come from the “S1200 Subject” release (see https://www.humanconnectome.org/data). A total of 954 participants were selected for this study. See Table 1 for basic information about the participants. Due to the lack of accuracy of gambling, social and motor behaviors, only the results of task working memory (TWmemory), language, emotion, and relationship are considered. All participants were assessed for a history of neurological and psychiatric disorders, psychotropic drug use, and physical condition or implants.

**Table 1:** Basic information of the participants.

**Characteristics**	**Value**
Gender (Male: Female)	503: 451
Age (Years)	22–35
Language accuracy	80.03 ± 6.96
Emotion accuracy	97.50 ± 3.65
Relational accuracy	76.26 ± 12.49
TWmemory accuracy	87.79 ± 8,79
Fluid intelligence	107.31 ± 16.60
Crystallized intelligence	111.10 ± 16.61

### Data and Data Pre-processing

The whole-brain echoplanar scans were acquired with a 32-channel head coil on a modified 3T Siemens Skyra with repetition time (TR) = 720 ms, echo time (TE) = 33.1 ms, flip angle = 52°, bandwidth (BW) = 2290 Hz/Px, in-plane field of view (FOV) = 208 × 18 mm, 72 slices, 2.0-mm isotropic voxels, with a multi-band acceleration factor of 8 (Ugurbil et al., 2013). In addition to the resting state, there were seven in-scanner tasks designed to engage a variety of cortical networks related to emotion perception, relational reasoning, language processing, TWmemory, gambling, social cognition, and motor responses. Table 2 briefly introduces these seven tasks. Barch et al. (2013) introduced more detail about the tasks.

**Table 2:** Seven human connectome project fMRI tasks.

**Task**	**Time (min:s)**	**Task design**
Language	3:57	Participants were asked to answer questions, including story conditions and math conditions.
Emotion	2:16	Participants were asked to choose the same face at the bottom of the screen and the top of the screen.
Relational	2:56	Participants determine whether the shape, texture, and size of the two objects are the same.
Social	3:27	Participants watch video clips of objects interacting in an agentive way or random way.
Working memory	5:01	Participants respond when the picture shown on the screen is the same as the two trials back (2-back) or the same as the one shown at the start of the block (0-back).
Gambling	3:12	Participants guess whether the numbers on the card are greater than 5, to determine whether they will win or lose
Motor	3:34	Participants move their fingers, toes, or tongue according to the prompts

We used a minimally pre-processed version of the data that included spatial normalization to a standard template, motion correction, slice timing correction, intensity normalization, and surface and parcel constrained smoothing of 2 mm full width at half maximum (Glasser et al., 2013). Filtering 0.06–0.125 Hz was applied to the data, which was thought to be especially sensitive to dynamic changes in the task-related functional brain (Han et al., 2017). Since each task contained two runs, to avoid the impact of insufficient time on the experiment, both runs were connected to a BOLD signal. The first 10 time points and the last 10 time points were then removed to minimize the boundary effect (Ponce-Alvarez et al., 2015). To facilitate the comparison between resting and task-based conditions, both sets of data were identically processed.

### Definition of RSNs From Functional Imaging Data

We extracted the BOLD signal from the Human Connectome Project Multimodal Parcellation (HCP–MMP) atlas and used the BOLD signal for regional analysis. Using multimodal segmentation of the human cerebral cortex (Glasser et al., 2016), 360 brain regions were assigned to 14 RSNs, including visual network (VIS), somatomotor network (SMN), cingulo–opercular network (CON), premotor1 (PREM1), premotor2 (PREM2), default mode network (DMN), frontoparietal network (FPN), primary auditory (AUD), language (LAN), posterior cingulate (PCC), dorsal attention network (DAN), hippocampal (HIPP), posterior multimodal (PMULTI) and uncertain (NONE). Refer to Supplementary Figure 1 for the RSNs distribution.

### Calculate the Synchrony

The synchrony was used to describe the “instantaneous” collective behavior of a group of phase oscillators (Skardal and Arenas, 2020). Given that the interaction matrix transfers more behaviorally relevant information than isolating the functional connectivity between regions, an interaction matrix was estimated for each subject, reflecting the synchrony interaction of 14 RSNs (Alderson et al., 2018). First, converted the BOLD signal into a complex phase through the Hilbert transform. The calculation formula for synchrony was as follows:


$$Synchrony\;=\;\frac{1}{NT}\left|\sum_{t\;=\;1}^{T}\sum_{n\;=\;1}^{N}e^{i\theta_{n}(t)}\right|$$

where Θ_*n*_(*t*) is the instantaneous phase of oscillator *n* at time *t*, *T* is the number of time points. For the global synchrony, *N* is the number of brain regions. For the synchrony between the RSNs, each interaction involved two RSNs, where *N* refered to the number of brain regions included in the two RSNs. With the complete independence, all phases were uniformly distributed and synchrony approaches 0. Conversely, if all phases are equivalent, synchrony approaches 1 (Bakhshayesh et al., 2019; Zirkle and Rubchinsky, 2020).

### Network-Based Statistic

The NBS is a non-parametric statistical test designed to deal with the multiple comparisons problem in a graph by identifying the largest connected sub-component in topological space while controlling the family-wise error rate (FWER). The synchrony interaction matrix between the resting state and the task states of each subject was calculated, and the NBS test was carried out to determine the difference of synchrony between the resting state and the seven task states (Figure 1B).

### Principal Component Analysis

The PCA was the most common method to reduce the linear dimensionality (Wong et al., 2021). Its goal was to map high-dimensional data to low-dimensional space through a certain linear projection (Li J et al., 2019). Loadings were used for the importance of a variable in each principal component (PC), that is, the weight of each variable in different PCs how many (Petersen et al., 2016; Min et al., 2018). To quantify the level of sharing of the seven task-based configurations, the PCA was used to reduce the dimensions of the seven task-based synchrony interaction matrices to a one-dimensional matrix, retaining most of the characteristics of the task states (Figure 1B). The synchrony interaction matrix of a single subject was vectorized based on the seven task states and obtained the task architecture of the synchrony of a single subject by PCA. Subsequently, a task-general architecture of synchrony was obtained by simple average across subjects.

### Update Efficiency

Update efficiency refers to the ability to switch from a resting state into a task-based configuration (Schultz and Cole, 2016a). The high update efficiency indicates that the network configuration was similar between the resting and task state, and few changes are required to complete the switch from the resting state to the task state. The high difference in network configuration between resting state and task state corresponds to the low update efficiency, suggesting that many changes were required to achieve task switching. The update efficiency was calculated for all subjects by vectorizing the upper triangular half and diagonal of the resting and task-general architecture of synchrony and calculating their Pearson correlation coefficient. Finally, the update efficiency of all subjects was transformed to a normal distribution by Fisher's z-distribution.

## Results

### Difference of Global Synchrony Between Resting State and Task States

**Figure 2:**
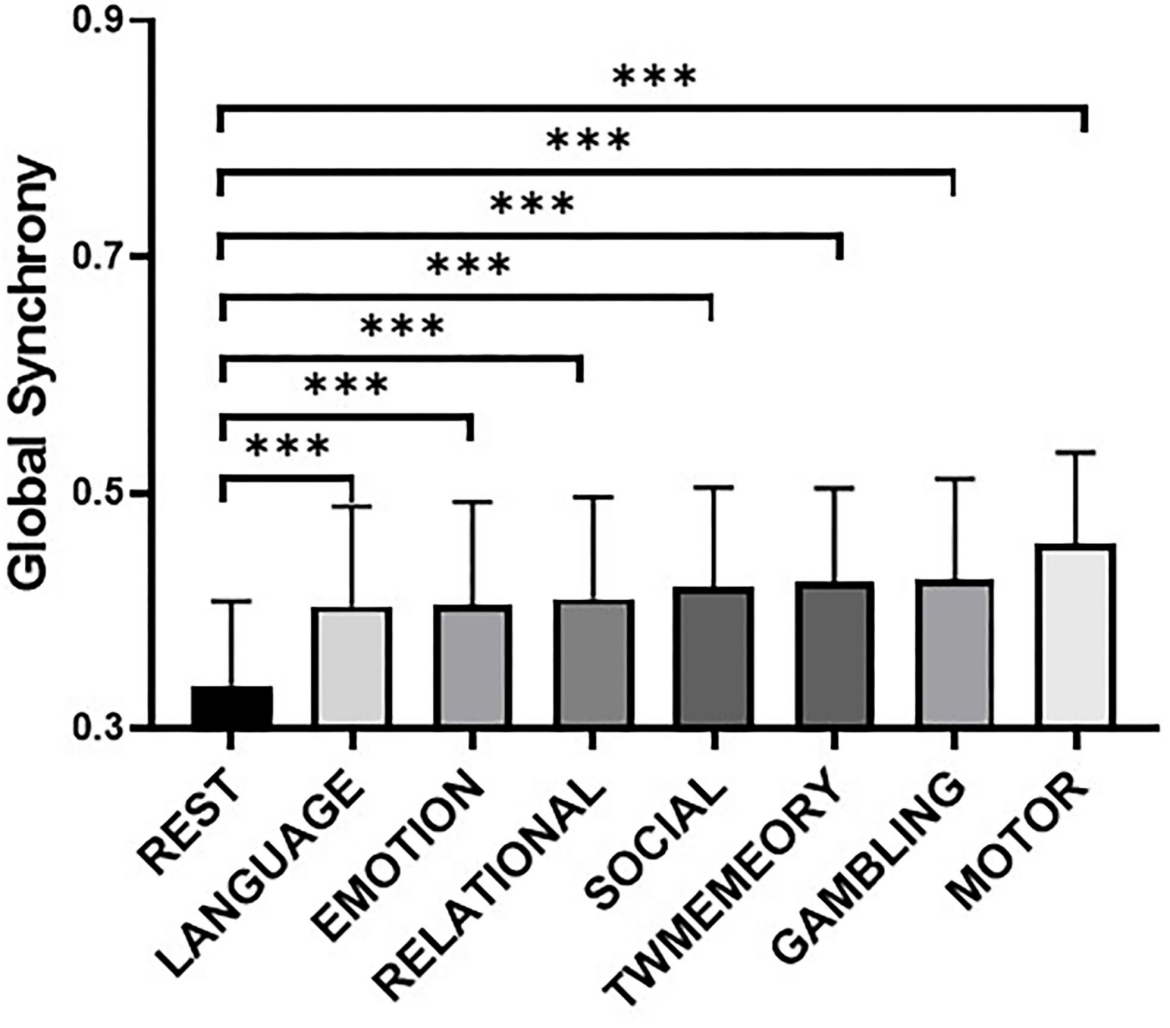
The difference of global synchrony during resting state and different task states. Bars display the mean value, 95% CI, and one SD with individual subjects indicated (***, *p* < 0.001). Tasks are arranged in ascending order of mean synchrony.

Compared with the resting state, the global synchrony during the tasks was significantly higher (Figure 2). These mainly included language (*t* = 19.01, *p* < 0.001), emotion (*t* = 19.06, *p* < 0.001), relational (*t* = 20.67, *p* < 0.001), social (*t* = 23.49, *p* < 0.001), TWmemory (*t* = 25.35, *p* < 0.001), gambling (*t* = 25.04, *p* < 0.001), and motor (*t* = 36.09, *p* < 0.001).

### Difference of RSNs Synchrony Between Resting State and Task States

**Figure 3:**
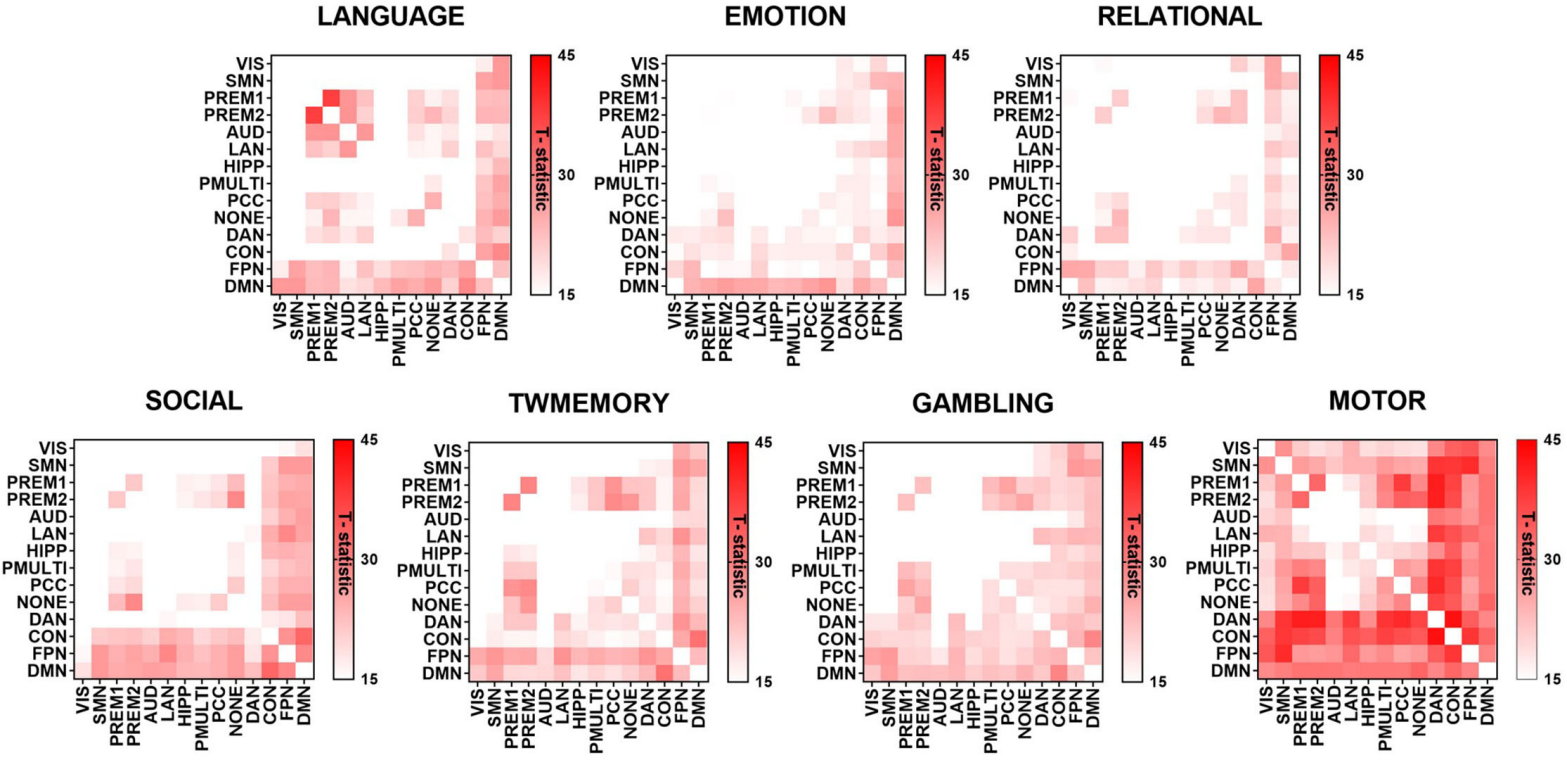
The difference of synchrony between RSNs in resting state and different task states. The graph used the t-value to show the largest connected subgraph of the difference. The darker the color, the greater the difference.

Figure 3 showed the maximum connected subgraph with increased synchrony detected by NBS under a fixed threshold of seven tasks. A fixed threshold of 15 was selected to visualize the increase in synchrony between RSNs under the same scale (*p* < 0.001, *corrected*). Across the seven tasks, the increase in synchrony was consistent. In addition, the increase of synchrony in high-order cognitive networks was more extensive (see Supplementary Figure 2).

### The Similarity of RSNs Synchrony During the Tasks

**Figure 4:**
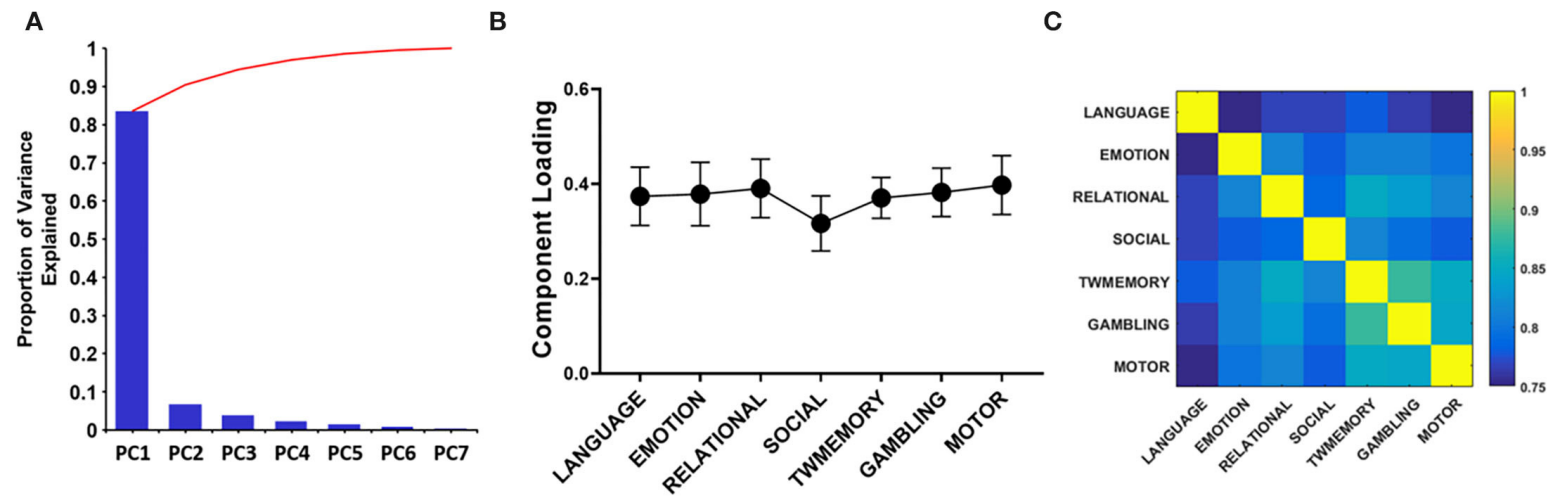
The PCA quantifies the degree of sharing based on the functional configuration of seven tasks. **(A)** The histogram of each component accounts for the variance between the seven tasks, and the broken line indicates the cumulative proportion of each component. **(B)** The loading of seven tasks. The error bar indicates the standard deviation. **(C)** Correlation between the synchrony of the seven tasks.

Through PCA, there were similar results among the seven tasks. On average, the PC1 accounted for 85% of the variance (Figure 4A). The loadings of the seven tasks were positive and evenly distributed (Figure 4B). These included the following: Language = 0.374, emotion = 0.378, relational = 0.390, social = 0.316, TWmemory = 0.370, gambling = 0.382, and motor = 0.397. The Pearson correlation was performed on the synchrony between seven tasks, it found that the correlation value can reach more than 0.75 (*p* < 0.01; Figure 4C).

### High Synchrony and Low Synchrony Between RSNs

**Figure 5:**
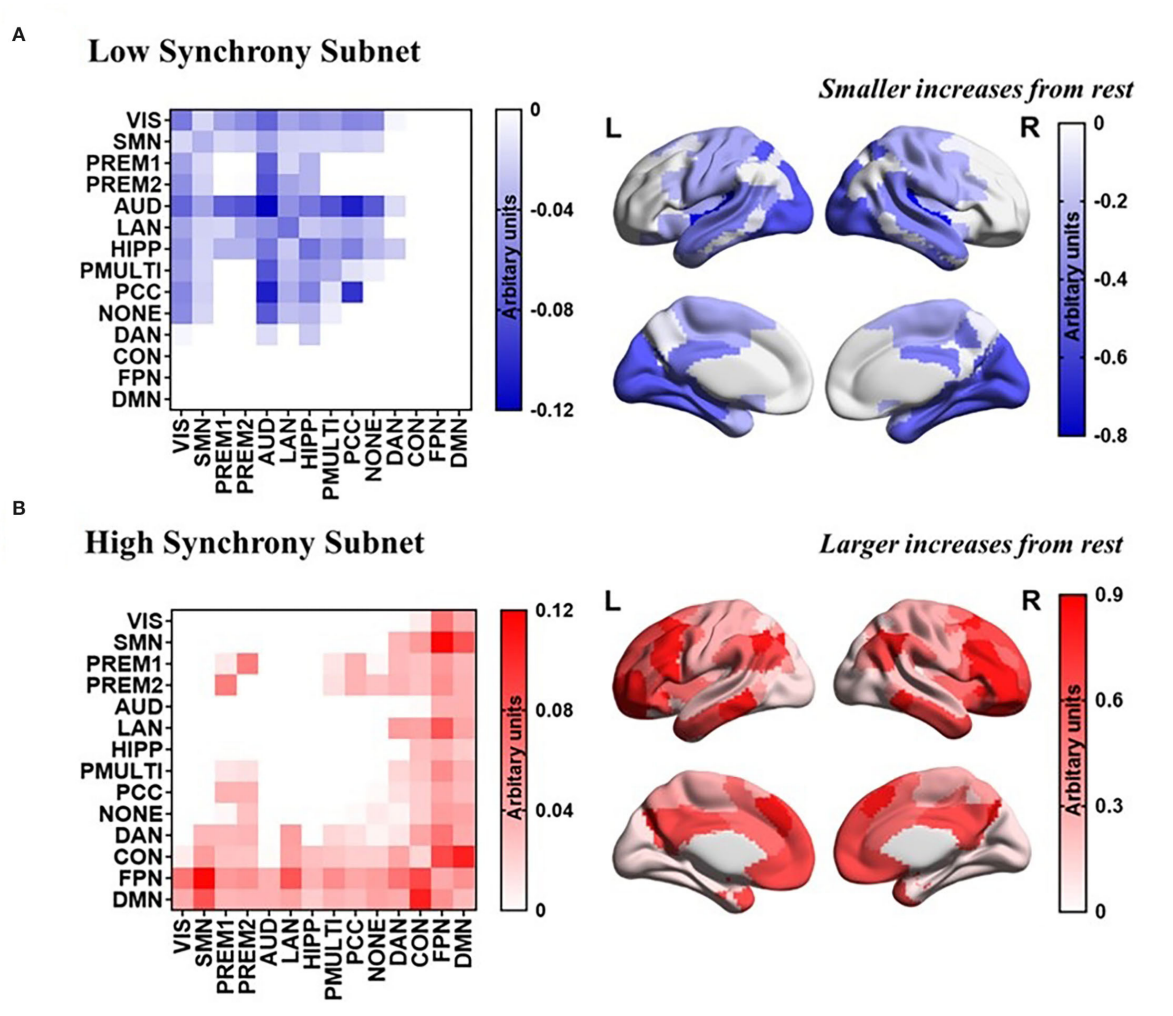
PCA reveals a task-general network architecture of synchrony. **(A)** Low synchrony subnet. **(B)** High synchrony subnet.

The task-general architecture of synchrony was decomposed into a low synchrony subnet (Figure 5A) and a high synchrony subnet (Figure 5B). The high synchrony subnets mainly included high-order cognitive networks, while low synchrony subnets were related to sensorimotor networks.

### Correlation Between Synchrony of RSNs and Update Efficiency

**Figure 6:**
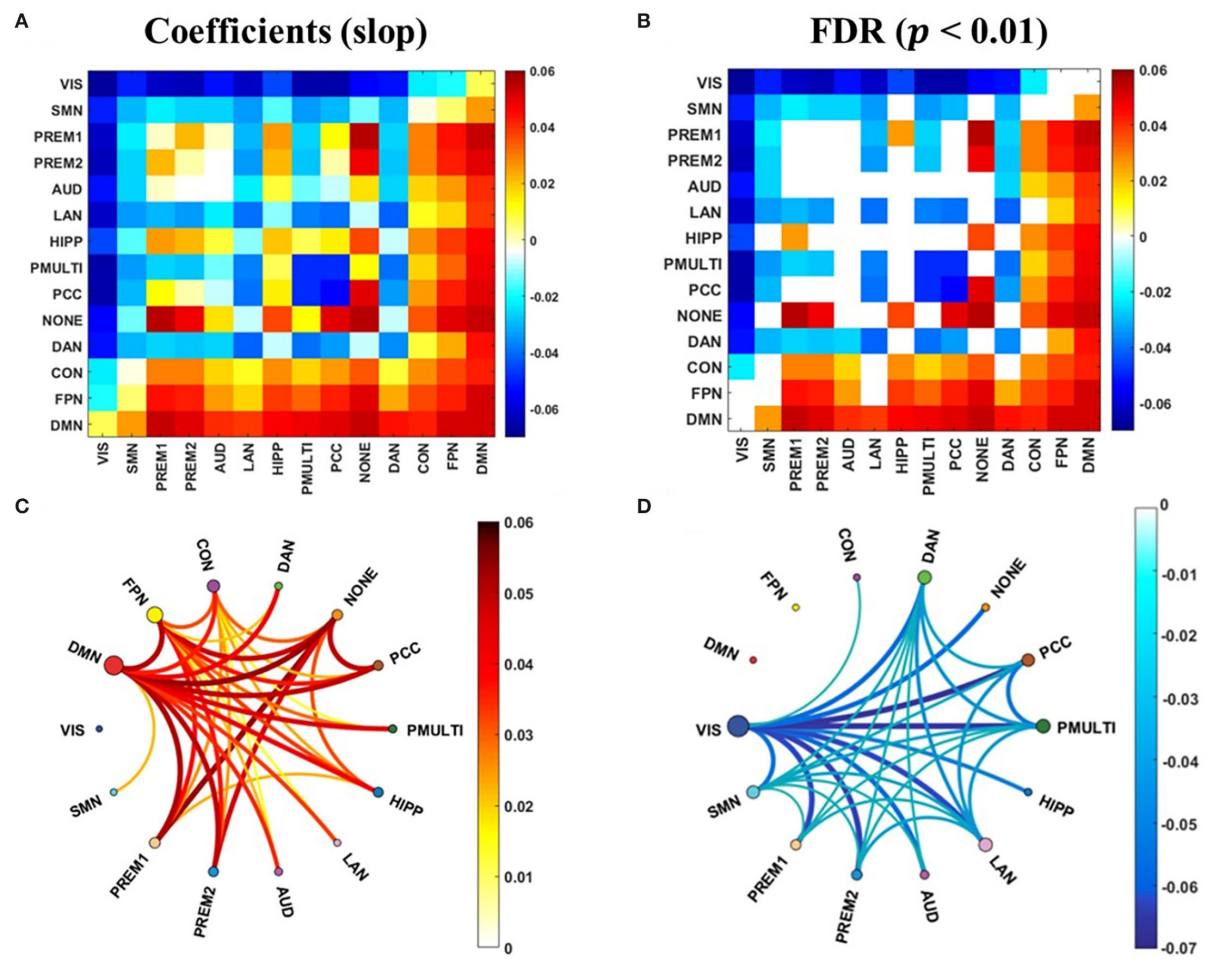
The correlation between the synchrony of the RSNs and the update efficiency. **(A)** The correlation coefficient (slope) between synchrony of RSNs and update efficiency. **(B)** There is a significant correlation coefficient between synchrony of RSNs and update efficiency. **(C)** There are RSNs with a positive correlation between update efficiency and synchrony. The darker the color, the higher the correlation. The size of the node represents the sum of the correlations at the RSN. **(D)** There are RSNs with a negative correlation between update efficiency and synchrony.

The correlation analysis between the synchrony and the update efficiency of the resting state and the task states network was carried out respectively. The results showed that there is no significant correlation between the synchrony of the task states network and the update efficiency. The synchrony of the RSNs and update efficiency were significantly correlated, and the linear regression analysis was performed to obtain the slope of the linear regression equation (Figure 6A). And after the false discovery rate (FDR) correction, it has a significant correlation (Figure 6B, *p* < 0.01). The RSNs showing a significant positive correlation was mainly in high-order cognitive networks (Figure 6C), while the most sensorimotor networks were negatively correlated (Figure 6D).

### Correlation Between Update Efficiency and Cognitive Performance

**Figure 7:**
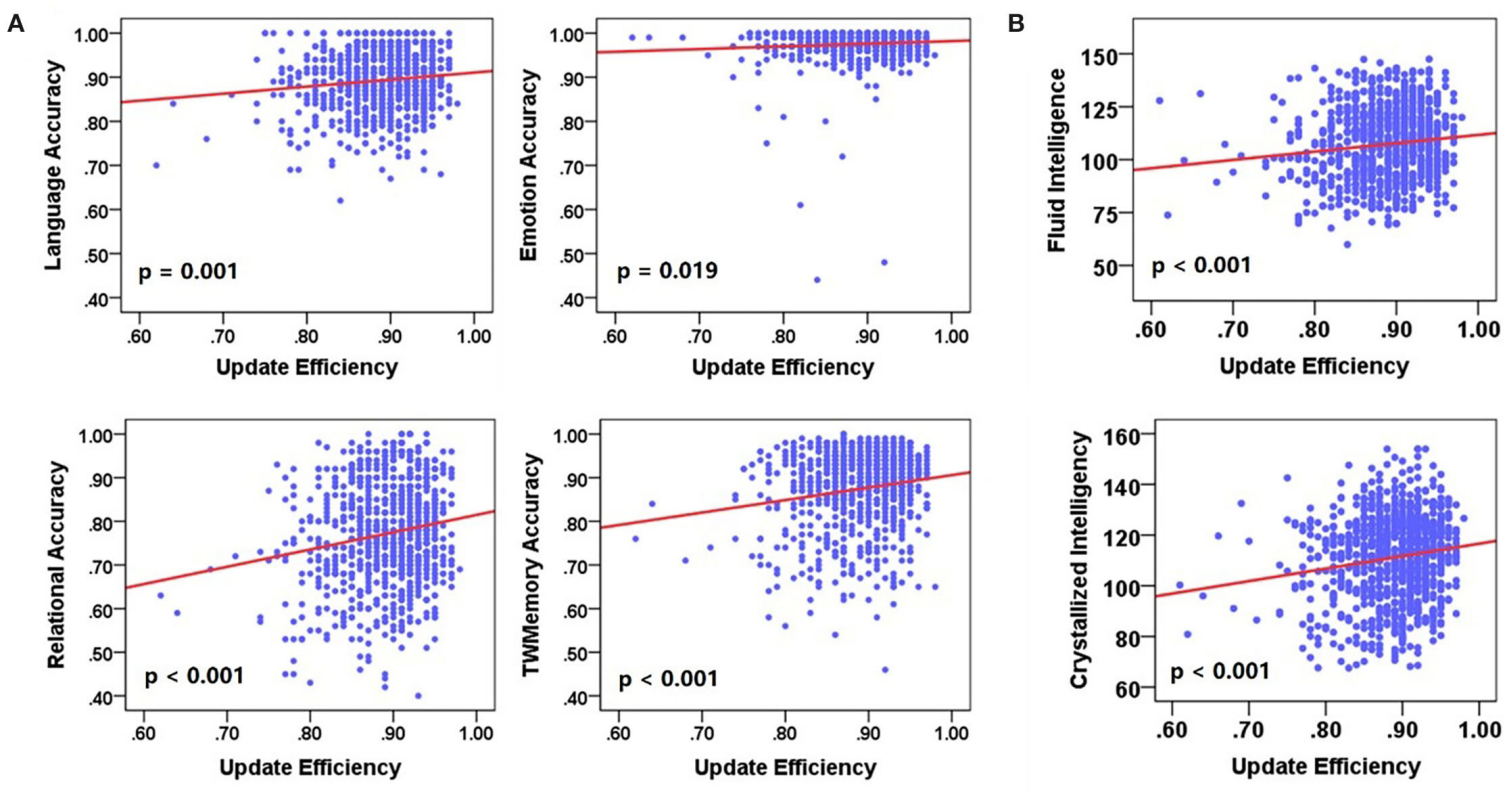
The correlation between update efficiency and cognitive performance. **(A)** The correlation between update efficiency and behavioral accuracy. **(B)** The correlation between update efficiency and cognitive intelligence. There is a positive correlation between update efficiency and behavioral accuracy.

Next, a linear regression analysis was performed on cognitive performance and update efficiency. There was a significant positive correlation between update efficiency and behavioral accuracy (Figure 7A) or cognitive intelligence (Figure 7B). Behavioral accuracy included language (*F* = 12.128, *p* = 0.001), emotion (*F* = 5.500, *p* = 0.019), relational (*F* = 24.073, *p* < 0.001), and work memory (*F* = 23.232, *p* < 0.001). The cognitive intelligence included the fluid intelligence (*F* = 13.787, *p* < 0.001) and crystalline intelligence (*F* = 21.732, *p* < 0.001).

## Discussion

The research mainly explored whether there was a task-general architecture of synchrony during the tasks. We studied 954 healthy young participants. During the tasks, the synchrony was higher than the resting state (Figure 2), especially in the higher order cognitive networks (Figure 3). Also, there were similar patterns for different tasks. Then we received a task-general architecture of synchrony by PCA (Figures 4, 5). In addition, the high synchrony of high-order cognitive networks in the resting state can promote task switching effectively (Figure 6). The participants with high update efficiency had better cognitive performance (Figure 7). The results are discussed in detail hereafter.

### There Was the Task-General Architecture of Synchrony Between Different Tasks

Our results proved that there was a “task-general architecture” in synchrony (Figures 4, 5). We reached the conclusion based on the following three results: (1) Most of the differences between tasks were resolved by PC1 (85%); (2) The loadings were evenly distributed; (3) There was a high correlation between the seven tasks. The current results are in agreement with previous studies, the functional connection network between different tasks had similar patterns (Schultz and Cole, 2016b; Chan et al., 2017). Also, the metastability showed a similar pattern for different tasks (Alderson et al., 2018). The similarities in functional network configurations across the different behavioral paradigms form the so-called “task-general architecture” (Cole et al., 2014). Then the proportion of changes in cerebral blood flow caused by different tasks was less than 5% (Raichle, 2010). It also showed from the side that different tasks have similar patterns.

The experience-dependent changes in the functional connectivity of the resting state demonstrate a certain plausibility for task-driven mechanisms (Hearne et al., 2017; Millar et al., 2021). The functional network architecture of the brain during the task performance was shaped, primarily, by an intrinsic network architecture that was also present during the resting state and, secondarily, by the evoked task-general and task-specific network changes (Raichle, 2010; Messel et al., 2019; Boring et al., 2020). Overall, the brain has a task-general architecture, but there were still specific task configurations in different tasks (Cole et al., 2014). This was the reason why there were similar but not identical patterns in seven tasks.

### The Synchrony of High-Order Cognitive Networks Has Increased More Widely During Tasks

The high-order cognitive networks had higher synchrony during the tasks, while the synchrony of the sensorimotor networks was relatively low (Figure 2). We suspected that the result was related to the following reasons. Both higher order cognitive networks tended to have high between-RSN connectivity, indicating their roles as connector RSNs. The close connection between these RSNs and the rest of the brain could form a mechanistic explanation for their utility in myriad complex cognitive processes (Gu et al., 2015a). In contrast to high-order cognitive networks that act as connectors, the sensorimotor networks tended to have poor between-RSN connectivity, indicating their roles as provincial RSNs. The weak connection between these RSNs and the rest of the brain indicated that they displayed distinct profiles of neurophysiological activity, and might perform more segregated functions (Gu et al., 2015b).

The previous studies had identified the exchange of information between regions was carried out through synchrony (Palmigiano et al., 2017; Li M et al., 2019). Then the high-order cognitive networks were global hubs (Meijer et al., 2017; Ferrier et al., 2020), and high-order cognitive networks played a disproportionate role in shaping information transfer between regions throughout the brain (Ito et al., 2017). So, the universal flexible hub networks showed a higher synchrony during the mission. The high-order cognitive networks indicate that it is more flexible to transfer task information across regions and networks (Ito et al., 2017).

### The High Synchrony of the Resting State High-Order Cognitive Networks Can Promote Task-Switching Ability

The high synchrony of the high-order cognitive networks coupling and the low synchrony of sensorimotor network coupling could promote the update efficiency between resting state and task-general architecture (Figure 6). In the brain, it was assumed that the communication between nerve groups was based on coherence. Through coherence, two neural combinations of synchronized activities can exchange information (Fries, 2005; Vasa et al., 2015). The update efficiency of synchrony was a method of isolation and integration. It indicated that during the resting state, the high degree of synchrony between the high-order cognitive networks can predict task performance, and network connectivity showed the tendency of integration and separation related to cognitive performance (Alderson et al., 2020). The exchange of information between the high-order cognitive networks is more flexible, the information exchange of sensorimotor networks is more stable, and the ability to switch from resting state to task configuration is stronger.

On the contrary, the changes in synchrony of the task-based RSNs have nothing to do with update efficiency. The brain activity at the resting state when subjects were not performing any explicit task predicted differences in fMRI activation across a range of cognitive paradigms. The resting state functional connectivity thus already contained the repertoire that is then expressed during task-based fMRI (Tavor et al., 2016). The observed fluctuations in network topology during the break were likely to be directly related to ongoing cognitive processing (Shine and Poldrack, 2018). The current research on RSNs showed that the functional couplings between regions at resting state contained information relevant to cognition, perception, and behavior (Sadaghiani and Kleinschmidt, 2013), rather than simply reflecting an invariant structural anatomy, historical co-activation patterns, or internal dynamics of local areas; the intrinsic activity predicted subsequent perceptual processing (van den Berg et al., 2016; Xu et al., 2021).

### Participants With Pre-configuration Showed a Better Performance

The update efficiency in brain network organization is positively related to general intelligence and behavior accuracy (Figure 7), the ability to perform a wide variety of cognitively challenging tasks well. It showed that the ability of participants to answer correct questions inside and outside the scanner is related to their internal neuron dynamics. The update efficiency reflected the difficulty of switching to the task-general architecture from the resting state (Schultz and Cole, 2016a,b). Specifically, the brain network configuration at the resting state was already closer to a wide variety of task configurations in intelligent individuals. The ability to modify network connectivity efficiently when task demands change is a hallmark of high intelligence (Cole et al., 2014).

Increasing reasoning demands were supported by the flexible reconfiguration of large-scale functional brain networks (Cocchi et al., 2014), but a recent study has demonstrated that such reconfigurations are relatively modest and occur within a preserved global network architecture (Hearne et al., 2017). A successful cognition was likely contingent on possessing an adequate a priori dynamic configuration before the onset of task-relevant stimuli, as opposed to simple *ad hoc* adjustments after the fact (Bolt et al., 2018). Therefore, the resting state activity may reflect the brain's predictive engagement with the environment (Sadaghiani and Kleinschmidt, 2013). Given that the resting state reflects the previous experience and the anticipation of likely future events, an RSN architecture “pre-configured” for the task is more in line with future cognitive requirements.

### The Task States Synchrony Was Higher Than the Resting State Synchrony

Compared with the resting state, the global synchrony during the task is higher, and the synchrony interaction between RSNs is also higher. Compared with the resting state, the co-activation network shows higher global efficiency, smaller average clustering coefficient, and lower modularity, which shows that the global information transmission and system during task execution are more effective between integrations (Maffei and Sessa, 2021). The global integration of the brain increases during the tasks, and as the neural activities of the internal brain system required for task execution become relevant, the functional connections between RSNs become stronger (Cohen and D'Esposito, 2016; Wei et al., 2022).

The change of synchrony is regulated by the needs of cognitive tasks and is a distinctive feature of the continuous activity of the human cortex (Palva et al., 2005). In general, the successful behavior depends on effective communication between brain regions. The communication between brain regions can be assessed by analyzing the synchrony. Moreover, the degree of synchrony between regions is more representative of changes in brain function intensity than the analysis of task activation to a certain extent (Hummel and Gerloff, 2005). Also, the task performance regulates functional interactions in the brain (Li et al., 2021). When performing a task, the brain adjusts its functional connections to exchange more information and reconfigure the brain network according to the task (Friston, 2011). Therefore, the synchrony is higher during the task.

## Conclusions

We reported a study about the synchrony of healthy young people at resting state and task states. The different tasks have a task-general architecture of synchrony. Compared to the resting state, the synchrony was significantly higher during the tasks, especially in high-order cognitive networks. It showed that synchrony provides great potential for associating brain activities with cognition and behavior. In addition, subjects with high synchrony at resting state have better pre-configurations and show more outstanding cognitive abilities.

## Data Availability Statement

The original contributions presented in the study are included in the article/Supplementary Material, further inquiries can be directed to the corresponding author/s.

## Ethics Statement

The studies involving human participants were reviewed and approved by Washington University–Minnesota Consortium Human Connectome Project. The patients/participants provided their written informed consent to participate in this study.

## Author Contributions

JX and CF: study design. LY, JL, and CF: data collection or acquisition. CF and XC: statistical analysis. JX, XC, and JW: interpretation of results. CF, XC, and JX: drafting the manuscript work, or revising it critically for important intellectual content. All authors contributed to the article and approved the submitted version.

## Funding

This project was supported by the National Natural Science Foundation of China (61873178), the Shanxi Provincial International Cooperation Foundation (201803D421047), and the Natural Science Foundation of Shanxi Province (20210302124550).

## Conflict of Interest

The authors declare that the research was conducted in the absence of any commercial or financial relationships that could be construed as a potential conflict of interest.

## Publisher's Note

All claims expressed in this article are solely those of the authors and do not necessarily represent those of their affiliated organizations, or those of the publisher, the editors and the reviewers. Any product that may be evaluated in this article, or claim that may be made by its manufacturer, is not guaranteed or endorsed by the publisher.

## References

[B1] Alderson T. H. Bokde A. L. W. Kelso J. A. S. Maguire L. Coyle D. (2020). Metastable neural dynamics underlies cognitive performance across multiple behavioural paradigms. Hum. Brain Mapp. 41, 3212–3234. 10.1002/hbm.25009 32301561 PMC7375112

[B2] Alderson T. H. Bokde A. L. W. Kelso J. A. S. Maguire L. Coyle D. Alzheimer's Disease Neuroimaging I. (2018). Metastable neural dynamics in Alzheimer's disease are disrupted by lesions to the structural connectome. Neuroimage 183, 438–455. 10.1016/j.neuroimage.2018.08.033 30130642 PMC6374703

[B3] Avena-Koenigsberger A. Misic B. Sporns O. (2017). Communication dynamics in complex brain networks. Nat Rev Neurosci 19, 17–33. 10.1038/nrn.2017.149 29238085

[B4] Bakhshayesh H. Fitzgibbon S. P. Janani A. S. Grummett T. S. Pope K. J. (2019). Detecting synchrony in EEG: a comparative study of functional connectivity measures. Comput. Biol. Med. 105, 1–15. 10.1016/j.compbiomed.2018.12.005 30562626

[B5] Barch D. M. Burgess G. C. Harms M. P. Petersen S. E. Schlaggar B. L. Corbetta M.. (2013). Function in the human connectome: task-fMRI and individual differences in behavior. Neuroimage 80, 169–189. 10.1016/j.neuroimage.2013.05.033 23684877 PMC4011498

[B6] Bolt T. Anderson M. L. Uddin L. Q. (2018). Beyond the evoked/intrinsic neural process dichotomy. Netw. Neurosci. 2, 1–22. 10.1162/NETN_a_00028 29911670 PMC5989985

[B7] Boring M. J. Ridgeway K. Shvartsman M. Jonker T. R. (2020). Continuous decoding of cognitive load from electroencephalography reveals task-general and task-specific correlates. J. Neural Eng. 17, 056016. 10.1088/1741-2552/abb9bc 32947265

[B8] Bullmore E. Sporns O. (2012). The economy of brain network organization. Nat. Rev. Neurosci. 13, 336–349. 10.1038/nrn3214 22498897

[B9] Chan M. Y. Alhazmi F. H. Park D. C. Savalia N. K. Wig G. S. (2017). Resting-state network topology differentiates task signals across the adult life span. J. Neurosci. 37, 2734–2745. 10.1523/JNEUROSCI.2406-16.2017 28174333 PMC5354325

[B10] Cocchi L. Halford G. S. Zalesky A. Harding I. H. Ramm B. J. Cutmore T.. (2014). Complexity in relational processing predicts changes in functional brain network dynamics. Cereb. Cortex 24, 2283–2296. 10.1093/cercor/bht075 23563963

[B11] Cohen J. R. D'Esposito M. (2016). The segregation and integration of distinct brain networks and their relationship to cognition. J. Neurosci. 36, 12083–12094. 10.1523/JNEUROSCI.2965-15.2016 27903719 PMC5148214

[B12] Cole M. W. Bassett D. S. Power J. D. Braver T. S. Petersen S. E. (2014). Intrinsic and task-evoked network architectures of the human brain. Neuron 83, 238–251. 10.1016/j.neuron.2014.05.014 24991964 PMC4082806

[B13] Dajani D. R. Odriozola P. Winters M. Voorhies W. Marcano S. Baez A.. (2020). Measuring cognitive flexibility with the flexible item selection task: from fmri adaptation to individual connectome mapping. J. Cogn. Neurosci. 32, 1026–1045. 10.1162/jocn_a_01536 32013686

[B14] Ferrier J. Tiran E. Deffieux T. Tanter M. Lenkei Z. (2020). Functional imaging evidence for task-induced deactivation and disconnection of a major default mode network hub in the mouse brain. Proc. Natl. Acad. Sci. USA. 117, 15270–15280. 10.1073/pnas.1920475117 32541017 PMC7334502

[B15] Freitas L. G. A. Bolton T. A. W. Krikler B. E. Jochaut D. Giraud A. L. Huppi P. S.. (2020). Time-resolved effective connectivity in task fMRI: psychophysiological interactions of co-activation patterns. Neuroimage 212, 116635. 10.1016/j.neuroimage.2020.116635 32105884

[B16] Fries P. (2005). A mechanism for cognitive dynamics: neuronal communication through neuronal coherence. Trends Cogn. Sci. 9, 474–480. 10.1016/j.tics.2005.08.011 16150631

[B17] Friston K. J. (2011). Functional and effective connectivity: a review. Brain Connect. 1, 13–36. 10.1089/brain.2011.0008 22432952

[B18] Glasser M. F. Coalson T. S. Robinson E. C. Hacker C. D. Harwell J. Yacoub E.. (2016). A multi-modal parcellation of human cerebral cortex. Nature 536, 171–178. 10.1038/nature18933 27437579 PMC4990127

[B19] Glasser M. F. Sotiropoulos S. N. Wilson J. A. Coalson T. S. Fischl B. Andersson J. L.. (2013). The minimal preprocessing pipelines for the Human Connectome Project. Neuroimage 80, 105–124. 10.1016/j.neuroimage.2013.04.127 23668970 PMC3720813

[B20] Gu S. Pasqualetti F. Cieslak M. Telesford Q. K. Yu A. B. Kahn A. E.. (2015a). Controllability of structural brain networks. Nat. Commun. 6, 8414. 10.1038/ncomms9414 26423222 PMC4600713

[B21] Gu S. Satterthwaite T. D. Medaglia J. D. Yang M. Gur R. E. Gur R. C.. (2015b). Emergence of system roles in normative neurodevelopment. Proc. Natl. Acad. Sci. USA. 112, 13681–13686. 10.1073/pnas.1502829112 26483477 PMC4640772

[B22] Han S. Zong X. Hu M. Yu Y. Wang X. Long Z.. (2017). Frequency-selective alteration in the resting-state corticostriatal-thalamo-cortical circuit correlates with symptoms severity in first-episode drug-naive patients with schizophrenia. Schizophr. Res. 189, 175–180. 10.1016/j.schres.2017.02.019 28236519

[B23] Hearne L. J. Cocchi L. Zalesky A. Mattingley J. B. (2017). Reconfiguration of brain network architectures between resting-state and complexity-dependent cognitive reasoning. J. Neurosci. 37, 8399–8411. 10.1523/JNEUROSCI.0485-17.2017 28760864 PMC6596866

[B24] Hummel F. Gerloff C. (2005). Larger interregional synchrony is associated with greater behavioral success in a complex sensory integration task in humans. Cereb. Cortex 15, 670–678. 10.1093/cercor/bhh170 15342429

[B25] Ito T. Kulkarni K. R. Schultz D. H. Mill R. D. Chen R. H. Solomyak L. I.. (2017). Cognitive task information is transferred between brain regions via resting-state network topology. Nat. Commun. 8, 1027. 10.1038/s41467-017-01000-w 29044112 PMC5715061

[B26] Kringelbach M. L. Deco G. (2020). Brain states and transitions: insights from computational neuroscience. Cell Rep. 32, 108128. 10.1016/j.celrep.2020.108128 32905760

[B27] Li J. Bolt T. Bzdok D. Nomi J. S. Yeo B. T. T. Spreng R. N.. (2019). Topography and behavioral relevance of the global signal in the human brain. Sci. Rep. 9, 14286. 10.1038/s41598-019-50750-8 31582792 PMC6776616

[B28] Li M. Han Y. Aburn M. J. Breakspear M. Poldrack R. A. Shine J. M.. (2019). Transitions in information processing dynamics at the whole-brain network level are driven by alterations in neural gain. PLoS Comput. Biol. 15, e1006957. 10.1371/journal.pcbi.1006957 31613882 PMC6793849

[B29] Li S. H. Graham B. M. Werner-Seidler A. (2021). Gender differences in adolescent sleep disturbance and treatment response to smartphone app-delivered cognitive behavioral therapy for insomnia: exploratory study. JMIR Form Res. 5, e22498. 10.2196/22498 33755029 PMC8075040

[B30] Maffei A. Sessa P. (2021). Event-related network changes unfold the dynamics of cortical integration during face processing. Psychophysiology 58, e13786. 10.1111/psyp.13786 33550632

[B31] Markett S. Jawinski P. Kirsch P. Gerchen M. F. (2020). Specific and segregated changes to the functional connectome evoked by the processing of emotional faces: a task-based connectome study. Sci. Rep. 10, 4822. 10.1038/s41598-020-61522-0 32179856 PMC7076018

[B32] Meijer K. A. Eijlers A. J. C. Douw L. Uitdehaag B. M. J. Barkhof F. Geurts J. J. G.. (2017). Increased connectivity of hub networks and cognitive impairment in multiple sclerosis. Neurology 88, 2107–2114. 10.1212/WNL.0000000000003982 28468841

[B33] Messel M. S. Raud L. Hoff P. K. Skaftnes C. S. Huster R. J. (2019). Strategy switches in proactive inhibitory control and their association with task-general and stopping-specific networks. Neuropsychologia 135, 107220. 10.1016/j.neuropsychologia.2019.107220 31586553

[B34] Millar P. R. Ances B. M. Gordon B. A. Benzinger T. L. S. Morris J. C. Balota D. A. (2021). Evaluating cognitive relationships with resting-state and task-driven blood oxygen level-dependent variability. J. Cogn. Neurosci. 33, 279–302. 10.1162/jocn_a_01645 33135966 PMC7877897

[B35] Min W. Liu J. Zhang S. (2018). Edge-group sparse PCA for network-guided high dimensional data analysis. Bioinformatics 34, 3479–3487. 10.1093/bioinformatics/bty362 29726900

[B36] Palmigiano A. Geisel T. Wolf F. Battaglia D. (2017). Flexible information routing by transient synchrony. Nat. Neurosci. 20, 1014–1022. 10.1038/nn.4569 28530664

[B37] Palva J. M. Palva S. Kaila K. (2005). Phase synchrony among neuronal oscillations in the human cortex. J. Neurosci. 25, 3962–3972. 10.1523/JNEUROSCI.4250-04.2005 15829648 PMC6724920

[B38] Petersen A. Zhao J. Carmichael O. Muller H. G. (2016). Quantifying individual brain connectivity with functional principal component analysis for networks. Brain Connect. 6, 540–547. 10.1089/brain.2016.0420 27267074 PMC5084364

[B39] Ponce-Alvarez A. Deco G. Hagmann P. Romani G. L. Mantini D. Corbetta M. (2015). Resting-state temporal synchronization networks emerge from connectivity topology and heterogeneity. PLoS Comput. Biol. 11, e1004100. 10.1371/journal.pcbi.1004100 25692996 PMC4333573

[B40] Raichle M. E. (2010). Two views of brain function. Trends Cogn. Sci. 14, 180–190. 10.1016/j.tics.2010.01.008 20206576

[B41] Sadaghiani S. Kleinschmidt A. (2013). Functional interactions between intrinsic brain activity and behavior. Neuroimage 80, 379–386. 10.1016/j.neuroimage.2013.04.100 23643921

[B42] Schultz D. H. Cole M. W. (2016a). Higher intelligence is associated with less task-related brain network reconfiguration. J. Neurosci. 36, 8551–8561. 10.1523/JNEUROSCI.0358-16.2016 27535904 PMC4987432

[B43] Schultz D. H. Cole M. W. (2016b). Integrated brain network architecture supports cognitive task performance. Neuron 92, 278–279. 10.1016/j.neuron.2016.10.004 27764661

[B44] Shine J. M. Poldrack R. A. (2018). Principles of dynamic network reconfiguration across diverse brain states. Neuroimage, 180(Pt B), 396-405. 10.1016/j.neuroimage.2017.08.010 28782684

[B45] Skardal P. S. Arenas A. (2020). Higher order interactions in complex networks of phase oscillators promote abrupt synchronization switching. Commun. Phys. 3, 1–6. 10.1038/s42005-020-00485-0

[B46] Tavor I. Parker Jones O. Mars R. B. Smith S. M. Behrens T. E. Jbabdi S. (2016). Task-free MRI predicts individual differences in brain activity during task performance. Science. 352, 216–220. 10.1126/science.aad8127 27124457 PMC6309730

[B47] Ugurbil K. Xu J. Auerbach E. J. Moeller S. Vu A. T. Duarte-Carvajalino J. M.. (2013). Pushing spatial and temporal resolution for functional and diffusion MRI in the Human Connectome Project. Neuroimage 80, 80–104. 10.1016/j.neuroimage.2013.05.012 23702417 PMC3740184

[B48] van den Berg B. Appelbaum L. G. Clark K. Lorist M. M. Woldorff M. G. (2016). Visual search performance is predicted by both prestimulus and poststimulus electrical brain activity. Sci. Rep. 6, 37718. 10.1038/srep37718 27901053 PMC5128787

[B49] Van Essen D. C. Smith S. M. Barch D. M. Behrens T. E. Yacoub E. Ugurbil K.. (2013). The WU-Minn human connectome project: an overview. Neuroimage 80, 62–79. 10.1016/j.neuroimage.2013.05.041 23684880 PMC3724347

[B50] Vasa F. Shanahan M. Hellyer P. J. Scott G. Cabral J. Leech R. (2015). Effects of lesions on synchrony and metastability in cortical networks. Neuroimage 118, 456–467. 10.1016/j.neuroimage.2015.05.042 26049146

[B51] Wei J. Wang X. Cui X. Wang B. Xue J. Niu Y.. (2022). Functional integration and segregation in a multilayer network model of patients with schizophrenia. Brain Sci. 12:368. 35326324 10.3390/brainsci12030368 PMC8946586

[B52] Wong R. Luo Y. Mok V. C. T. Shi L. (2021). Advances in computerized MRIbased biomarkers in Alzheimer's disease. Brain Sci. Adv. 7, 26–43.

[B53] Xu S. Sun Q. Li M. Luo J. Cai G. Chen R.. (2021). Hippocampal restingstate functional connectivity with the mPFC and DLPFC moderates and mediates the association between education level and memory function in subjective cognitive decline. Brain Sci. Adv. 7, 124–140.

[B54] Zhang W. Tang F. Zhou X. Li H. (2020). Dynamic reconfiguration of functional topology in human brain networks: from resting to task states. Neural Plast. 2020, 8837615. 10.1155/2020/8837615 32963519 PMC7495231

[B55] Zhou X. Ma N. Song B. Wu Z. Liu G. Liu L.. (2021). Optimal organization of functional connectivity networks for segregation and integration with large-scale critical dynamics in human brains. Front. Comput. Neurosci. 15, 641335. 10.3389/fncom.2021.641335 33867963 PMC8044315

[B56] Zirkle J. Rubchinsky L. L. (2020). Spike-timing dependent plasticity effect on the temporal patterning of neural synchronization. Front. Comput. Neurosci. 14, 52. 10.3389/fncom.2020.00052 32595464 PMC7303326

