# Supplementary material for: The Task Pre-Configuration Is Associated With Cognitive Performance Evidence From the Brain Synchrony
Source: Front Comput Neurosci. 2022 May 6;16:883660. doi: 10.3389/fncom.2022.883660 (PMC9120823; doi:10.3389/fncom.2022.883660)
Supplement: Supplementary file 1 [file Data_Sheet_1.docx]

Supplementary Material


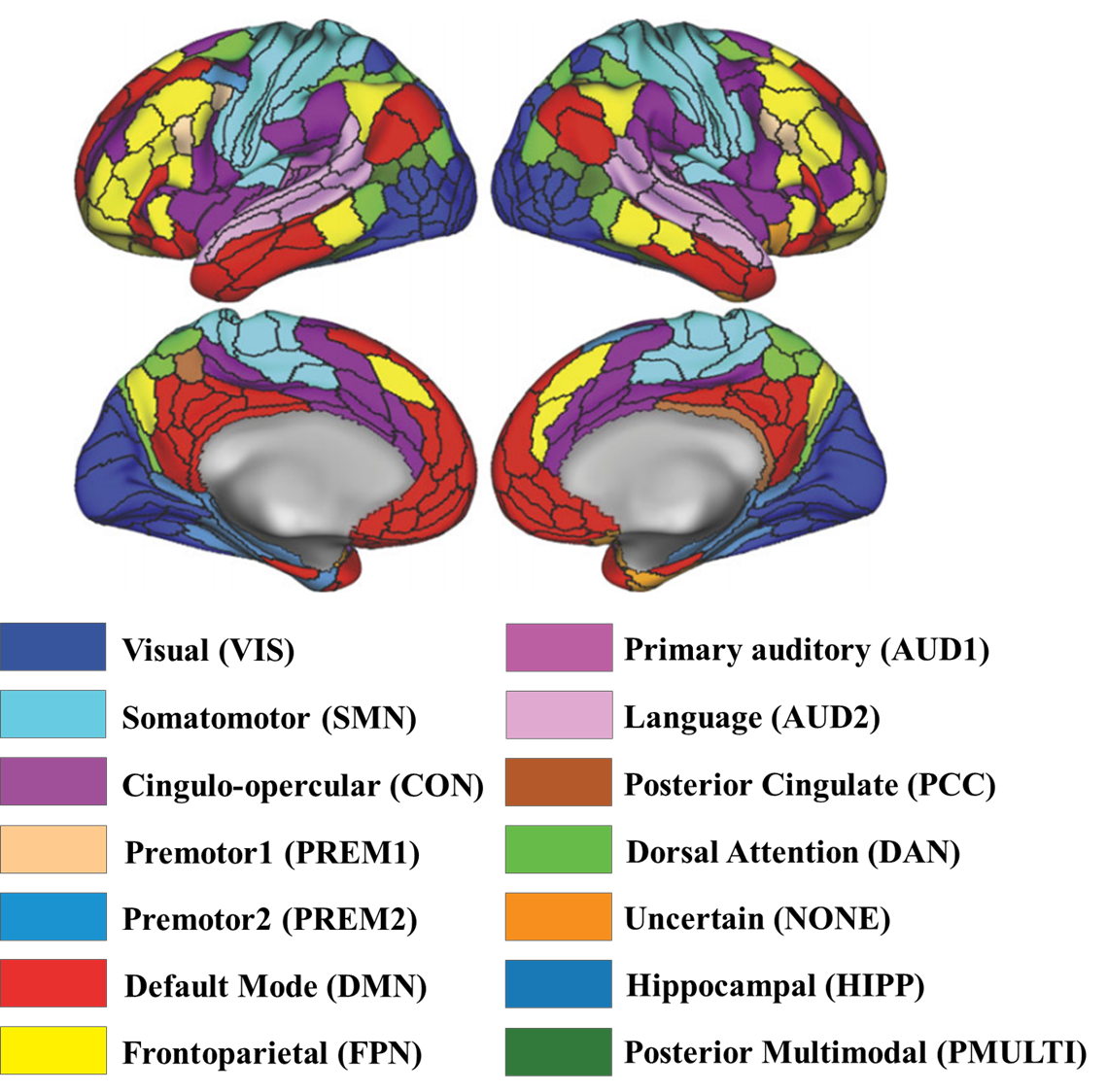


**Supplementary Figure 1.** The division of RSNs. The brain is divided into 14 RSNs, the same color representing the same RSN.

**Supplementary Figure 2.** The high-order cognitive networks displayed increased synchrony in their interactions with other RSNs across the seven tasks, these included cingulo-opercular network (CON), default mode network (DMN), frontoparietal network (FPN), dorsal attention network (DAN).
